# Autism and classification systems: a study of 84 children

**DOI:** 10.1186/1824-7288-36-10

**Published:** 2010-01-29

**Authors:** Matteo Chiappedi, Giorgio Rossi, Maura Rossi, Maurizio Bejor, Umberto Balottin

**Affiliations:** 1 Rehabilitation Unit, "Santa Maria alle Fonti" Medical Center, Don Carlo Gnocchi ONLUS Foundation, Salice Terme (PV), Italy; 2 Department of Neurological Sciences, University of Pavia, Pavia, Italy; 3 Child Neuropsychiatry Unit, IRCCS "C. Mondino" Foundation, Pavia, Italy; 4 Department of Surgical, Resuscitative, Rehabilitative and Transplant Sciences, University of Pavia, Pavia, Italy

## Abstract

**Background:**

A number of studies have shown that current classification systems (ICD 10, DSM IV TR) have limitation when applied to autistic children and the category PDD NOS (DSM IV TR) has in particular been criticized. To check the possible usefulness of other classification systems to better describe patient's functioning, we retrospectively studied 84 patients, seen consecutively in our Child Neurology and Psychiatry Department (excluding only those presenting for another disease even if with clinical signs of a PDD).

**Methods:**

We tried to classify them according to ICD 10, DSM IV TR, CFTMEA-R, "operational classification" (Manzano and Palacio) and de Ajuriaguerra's classification.

**Results:**

We found a good correspondence between DSM IV TR and ICD 10 and the use of psychodynamic classification systems (in particular CFTMEA-R) was useful to differentiate clinical subtypes collected under the PDD NOS etiquette according to DSM IV TR.

**Conclusions:**

To rationalize research efforts and find better tailored therapies, we need to improve PDD classification systems, using contributions coming from every field of child psychiatry and neurology: it's possible that 0-3 Classification could help this.

## Background

A number of studies have shown that current classification systems (ICD 10 [1], DSM IV TR [2]) have limitations when applied to children with Autism Spectrum Disorders. According to Cohen and Volkmar [3] classification systems should aim at improving communication, through their features (internal consistency, use easiness, good definition of categories) and being widely accepted. Cantwell [4] underlines that a classification system should provide a description unifying clinical, biochemical, genetical, neurophysiological and neuroimaging findings to identify specific categories with a unique natural history, prognosis and (if possible) therapy. This system should also be logical and easy to use in clinical settings. The term Autism Spectrum Disorders comes from the theoretical work of many Authors and includes a continuum moving from the "classical" autism described by Kanner, to Asperger's syndrome, to autistic-like forms and even to autistic traits in mental retardation [5].

Phenomenological polymorphism and a natural course not moving towards "normality" are among the main factors which make it difficult to reach a shared classification system. After the important contributions by Rutter and Ritvo, DSM III was the first system to use the Pervasive Developmental Disorders (PDD) category, which meant to be an a-theoretical definition including children with abnormalities interesting different areas of their development. Among the criticism ([6]; [7]), Golse [8] underlined the risk to lose the importance of the emotional aspects and to focus diagnosis and therapy only on presumed (but still unclear) organic factors. Anyway a strong rational reason to accept the PDD category was the possibility to unify all "autistic-like" syndromes, previously described under various names by different authors. Moreover, specific symptoms are usually present before 30 months of age, even if they are not always seen as relevant (and reported to the pediatrician) by parents.

Current international diagnostic systems (ICD 10 and DSM IV TR) have received different criticism; among them:

• the inclusion of Rett syndrome is questioned, given its established genetical origin;

• even if ICD 10 had been projected to use criteria compatible with (at that point "soon to come") DSM IV, it provides much more detailed research criteria;

• Atypical Autism is apparently well described, but these criteria are sometimes hard to use in clinical settings;

• PDD NOS are not well defined in terms of what should be excluded from their definition;

• a dimensional vision has been hypothesized to be potentially more useful than a categorical one;

• the distinction between Childhood Autism and PDD NOS in terms of functioning is unclear;

• the lack of well defined inclusion and exclusion criteria leads to a description of PDD NOS as something totally disconnected from Childhood Autism;

• it is possible that at least some PDD NOS are the evolution of a Childhood Autism (Towbin, 1996), given that PDD NOS diagnosis is usually delayed in time probably because of the less striking behavioral features;

• current classification seem to convey the idea that PDD are the result of some organic deficit, even when this is not evident.

Other classification systems have been proposed. In our Department, we have long been familiar with three of them: CFTMEA-R [9], de Ajuriaguerra's classification [10] and Manzano and Palacio's "operational classification" [11].

We also want to quote Multiple Complex Developmental Disorder (MCDD, described by Towbin et alii [12]), in which the behavior is strikingly similar to that seen in "psychotic disharmonies".

We wanted to check the possible usefulness of other classification systems to better describe patient's functioning. We used the three psychodynamic systems which were widely used before the introduction of DSM IV in Italy (in our Department, for instance, the difference of diagnostic system used in a retrospective analysis proved to favour psychodynamic systems before 1997 and ICD or DSM IV later on (*X *square test, P < 0.0001)).

## Patients and Methods

We reviewed medical records of 84 children, seen in our Department of Child Neurology and Psychiatry. Inclusion criteria were:

• less than 18 year old;

• being sent with a suspected PDD without an organic disorder diagnosed or suspected;

• being sent for a complete evaluation (i.e. not for specific problems such as epilepsy).

We had performed different evaluation protocols, according to the specific patient's needs. The main diagnostic instruments were:

• anamnesis (100% of patients);

• clinical examination (100% of patients);

• neurological examination (100% of patients);

• CARS (100% of patients);

• BSE (ERC-A-III; 100% of patients);

• development evaluation using Griffiths Mental Development Scales (59.5% of patients, the others being non reliable);

• play observations (at least 2 session, often video recorded; 100% of patients);

• blood and urine exams (including thyroid function (46,4%), plasma amino acids/plasma and urine organic acids (52,4%));

• genetic testing (karyotype 100%, Fragile X testing 51.2%);

• EEG (67.9%);

• evoked potentials (Visual Evoked Potentials and Brainstem Auditory Evoked Potentials, 71.4%);

• MRI (54.8%) or CT (34.5%) or both (4.8%).

We reviewed all available data in order to classify patients according to different systems:

• DSM IV TR;

• ICD 10;

• CFTMEA-R;

• Manzano and Palacio's "operational classification"

• De Ajuriaguerra's classification.

Due to space limitations, we will summarize here only the most important data in the anamnesis of our patients. They were mainly males (84.5%), and about half of them (52.8%) had been seen before 48 months of age (but 77.4% had been first seen by a specialist by the age of three). We tried to excluded, as described, patients sent to our Centre for a specific organic disorder; still one of our patients, sent with a provisional diagnosis of "idiopathic autism", was found to be affected by Sclerosis Tuberosa after brain MRI showed almost specific lesions.

Speech delay or absence was the most commonly reported reason to consult a specialist (82.1%), followed by non specified "relational difficulties" (56%), behavioral problems (46.4%), sleeping disturbances (22.6%), a global developmental delay (16.1%) or feeding disturbances (6.0%); 90.5% of patients presented three or less of these symptoms.

We found 46.4% of patients with neurological "soft signs"; 84.5% being isolated at least "quite often"; 70.2% with stereotyped behavior; 86% with a development delay (among those we could reliably evaluate); no specific internal medicine abnormalities; 47.4% EEG abnormalities; 15% brainstem auditory evoked potential alterations; 12.1% visual evoked potential alterations; 11.7% specific neuroradiological abnormalities (29.9% non specific).

## Results

We found a good correspondence between DSM IV TR and ICD 10 (about 1/3 of our patients being diagnosed as Childhood Autism or Autistic Disorder and 2/3 PDD NOS or Atypical Autism). Considering that PDD NOS should be a residual category, the number of patients receiving this diagnosis is obviously much higher than expected.

The diagnosis according to the different systems used is shown in details in Table 1 and 2. For CFTMEA-R and "operational classification" (Manzano and Palacio) only diagnostic categories attributed to at least one of our patients are shown. We were not able to classify 3 of our patients according to the "operational classification" (Manzano and Palacio), because we missed some important and specific information.

**Table 1:** Patients with Autistic Disorder (DSM IV TR criteria) classified according to CFTMEA-R, Manzano-Palacio "operational classification" and De Ajuriaguerra's classification

PATIENT	CFTMEA-R	Manzano-Palacio	De Ajuriaguerra
1	Kanner's Autism	Primary Autism	Kanner's early autistic psychosis

2	Kanner's Autism	Primary Autism	Kanner's early autistic psychosis

3	Kanner's Autism	Primary Autism	Kanner's early autistic psychosis

4	Kanner's Autism	Primary Autism	Kanner's early autistic psychosis

5	Kanner's Autism	Primary Autism	Kanner's early autistic psychosis

6	Kanner's Autism	Primary Autism	Kanner's early autistic psychosis

7	Kanner's Autism	Primary Autism	Kanner's early autistic psychosis

8	Kanner's Autism	Primary Autism	Kanner's early autistic psychosis

9	Kanner's Autism	Primary Autism	Kanner's early autistic psychosis

10	Kanner's Autism	Primary Autism	Kanner's early autistic psychosis

11	Deficit psychosis	Deficit Psychosis	Early deficit psychosis

12	Kanner's Autism	Primary Autism	Kanner's early autistic psychosis

13	Other Autistic Disorders	Secondary Autism	Kanner's early autistic psychosis

14	Kanner's Autism	Primary Autism	Kanner's early autistic psychosis

15	Kanner's Autism	Primary Autism	Kanner's early autistic psychosis

16	Other Autistic Disorders	Secondary Autism	Kanner's early autistic psychosis

17	Kanner's Autism	Primary Autism	Kanner's early autistic psychosis

18	Deficit psychosis	Deficit Psychosis	Early deficit psychosis

19	Kanner's Autism	Primary Autism	Kanner's early autistic psychosis

20	Kanner's Autism	Primary Autism	Kanner's early autistic psychosis

21	Psychotic Dysarmonia	Disorganizing Psychosis	Early personality distortion

22	Kanner's Autism	Primary Autism	Kanner's early autistic psychosis

23	Kanner's Autism	Primary Autism	Kanner's early autistic psychosis

24	Kanner's Autism	Primary Autism	Kanner's early autistic psychosis

25	Kanner's Autism	Primary Autism	Kanner's early autistic psychosis

26	Kanner's Autism	Primary Autism	Kanner's early autistic psychosis

27	Deficit psychosis	Deficit Psychosis	Early deficit psychosis

28	Kanner's Autism	Primary Autism	Kanner's early autistic psychosis

29	Kanner's Autism	Primary Autism	Kanner's early autistic psychosis

30	Other Autistic Disorders	Primary Autism	Early deficit psychosis

**Table 2:** Patients with Pervasive Developmental Disorder Not Otherwise Specified (DSM IV TR criteria) classified according to CFTMEA-R, Manzano-Palacio "operational classification" and De Ajuriaguerra's classification

PATIENT	CFTMEA-R	Manzano-Palacio	De Ajuriaguerra
1	Other Autistic Disorders	Secondary Autism	Early personality distortion

2	Childhood Schizophrenic Disorders	Disorganizing Psychosis	Early personality distortion

3	Deficit psychosis	Disorganizing Psychosis	Early personality distortion

4	Other Autistic Disorders	Primary Autism	Early personality distortion

5	Psychotic Dysarmonia	Disorganizing Psychosis	Early personality distortion

6	Other Autistic Disorders	Secondary Autism	Early personality distortion

7	Other Autistic Disorders	Primary Autism	Early personality distortion

8	Other Autistic Disorders	Primary Autism	Early personality distortion

9	Psychotic Dysarmonia	Disorganizing Psychosis	Early personality distortion

10	Other Autistic Disorders	Primary Autism	Early personality distortion

11	Psychotic Dysarmonia	Unable to classify	Early personality distortion

12	Psychotic Dysarmonia	Symbiotic Psychosis	Early personality distortion

13	Psychotic Dysarmonia	Disorganizing Psychosis	Early personality distortion

14	Psychotic Dysarmonia	Disorganizing Psychosis	Early personality distortion

15	Deficit psychosis	Deficit Psychosis	Early deficit psychosis

16	Other Autistic Disorders	Primary Autism	Early personality distortion

17	Psychotic Dysarmonia	Disorganizing Psychosis	Early personality distortion

18	Psychotic Dysarmonia	Secondary Autism	Early personality distortion

19	Other Autistic Disorders	Primary Autism	Early deficit psychosis

20	Childhood Schizophrenic Disorders	Disorganizing Psychosis	Early personality distortion

21	Kanner's Autism	Primary Autism	Kanner's early autistic psychosis

22	Psychotic Dysarmonia	Secondary Autism	Early personality distortion

23	Deficit psychosis	Deficit Psychosis	Early deficit psychosis

24	Deficit psychosis	Deficit Psychosis	Early deficit psychosis

25	Psychotic Dysarmonia	Symbiotic Psychosis	Early personality distortion

26	Other Autistic Disorders	Secondary Autism	Kanner's early autistic psychosis

27	Other Autistic Disorders	Primary Autism	Kanner's early autistic psychosis

28	Psychotic Dysarmonia	Symbiotic Psychosis	Early personality distortion

29	Deficit psychosis	Deficit Psychosis	Early personality distortion

30	Kanner's Autism	Primary Autism	Kanner's early autistic psychosis

31	Deficit psychosis	Deficit Psychosis	Early deficit psychosis

32	Deficit psychosis	Deficit Psychosis	Early deficit psychosis

33	Psychotic Dysarmonia	Disorganizing Psychosis	Early personality distortion

34	Psychotic Dysarmonia	Symbiotic Psychosis	Early personality distortion

35	Psychotic Dysarmonia	Unable to classify	Early personality distortion

36	Other Autistic Disorders	Secondary Autism	Early personality distortion

37	Other Autistic Disorders	Secondary Autism	Early personality distortion

38	Other Autistic Disorders	Secondary Autism	Early personality distortion

39	Other Autistic Disorders	Primary Autism	Early personality distortion

40	Other Autistic Disorders	Symbiotic Psychosis	Early personality distortion

41	Deficit psychosis	Deficit Psychosis	Early deficit psychosis

42	Psychotic Dysarmonia	Unable to classify	Early personality distortion

43	Psychotic Dysarmonia	Disorganizing Psychosis	Early personality distortion

44	Deficit psychosis	Deficit Psychosis	Early deficit psychosis

45	Other Autistic Disorders	Primary Autism	Kanner's early autistic psychosis

46	Other Autistic Disorders	Secondary Autism	Early personality distortion

47	Other Autistic Disorders	Secondary Autism	Early personality distortion

48	Deficit psychosis	Deficit Psychosis	Early deficit psychosis

49	Other Autistic Disorders	Secondary Autism	Early personality distortion

50	Other Autistic Disorders	Secondary Autism	Early personality distortion

51	Other Autistic Disorders	Primary Autism	Early deficit psychosis

52	Other Autistic Disorders	Secondary Autism	Early personality distortion

53	Kanner's Autism	Primary Autism	Kanner's early autistic psychosis

54	Other Autistic Disorders	Primary Autism	Early personality distortion

## Discussion

We found a good level of homogeneity (76.7%) among patients with a diagnosis of Autistic Disorder (according to DSM IV TR criteria) or Childhood Autism (according to ICD 10). They generally resemble Kanner's historical description of autistic children, even if a few less typical clinical pictures are included. The latters have more symptoms on all three core Autism Spectrum Disorders domains compared to those with Pervasive Developmental Disorders Not Otherwise Specified (PDD NOS), exactly as reported in current scientific literature [13].

In contrast, in the PDD NOS group (according to DSM IV TR, but superimposable to Atypical Autism group according to ICD 10) we found a low level of homogeneity (5.6%). However, the use of psychodynamic classification systems (in particular CFTMEA-R) allowed to differentiate different clinical subtypes collected under the PDD NOS etiquette according to DSM IV TR (or Atypical Autism according to ICD 10). Putting together the psychodynamic classification systems, we found two main subgroups among our patients. They are defined on the basis of the prevalent symptoms: one has isolation, the hallmark of autism according to common speaking, as the main clinical feature; the other is characterized by developmental delay, which is generally early evidenced by parents. These two main factors obviously interact with each other and with many others to create the "autistic spectrum". Groups with coherent diagnosis (and clinical features) are anyway too small to draw any definite conclusion.

Some patients with PDD NOS are included in the Primary Autism or Secondary Autism groups according to Manzano and Palacio's "operational classification". This is a consequence of the different definitions used.

Our study is limited by the implicit selection of our patients (as we work in a third level centre) and their age (only 52.4% were less the 4 year old). Another relevant limitation is due to its retrospective design.

We did not use the 0-3 Classification [14] either, because it has really specific features which had only in part been explored in the medical records we used.

## Conclusions

Recent scientific literature claims that to allow a systematic evaluation of existing classification systems, more comparative studies on one and the same sample of patients using different classification systems for the purposes of diagnostic codification are needed [15].

In order to rationalize research efforts and to find better tailored therapies, we need to improve autism classification systems, using contribution coming from every field of child psychiatry and neurology. Obviously this implies the ability to leave prejudices coming from all different sides. After Kandel's fundamental papers ([16,17]), we believe it's possible and necessary to re-open (or maybe create ex novo) a constructive dialogue between different models and views in order to serve children's best interest. This is in line with the suggested need for other approaches in the definition of Autism Spectrum Disorders subtypes, such as the dimensional one [13]. We can argue that the 0-3 Classification could be an effective and acceptable model for this integration, being at the same time consistent from a psychodynamic point of view and empirically based [18].

## Competing interests

The authors declare that they have no competing interests.

## Authors' contributions

MC, GR, MR and UB participated in study design and patient's clinical assessment. MB participated in the design of the study and performed data analysis. All authors contributed to draft the manuscript, which they all read and approved in the final version.
